# Tensile modulus of human orbital wall bones cut in sagittal and coronal planes

**DOI:** 10.1371/journal.pone.0259363

**Published:** 2021-11-05

**Authors:** Krzysztof Zerdzicki, Pawel Lemski, Pawel Klosowski, Andrzej Skorek, Marcin Zmuda Trzebiatowski, Mateusz Koberda

**Affiliations:** 1 Faculty of Civil and Environmental Engineering, Gdansk University of Technology, Gdansk, Poland; 2 Medical University of Gdańsk, Department of Otolaryngology, Gdańsk, Poland; 3 Medical University of Gdansk, Department of Ophtalmology, Gdansk, Poland; University of Life Sciences in Lublin, POLAND

## Abstract

In the current research, 68 specimens of orbital superior and/or medial walls taken from 33 human cadavers (12 females, 21 males) were subjected to uniaxial tension untill fracture. The samples were cut in the coronal (38 specimens) and sagittal (30 specimens) planes of the orbital wall. Apparent density (*ρ*_*app*_), tensile Young’s modulus (*E*-*modulus*) and ultimate tensile strength (UTS) were identified. Innovative test protocols were used to minimize artifacts and analyze the obtained data: (1) grips dedicated to non-symmetrical samples clamping were applied for mechanical testing, (2) non-contact measuring system of video-extensometer was employed for displacement registration, (3) ink imprint technique coupled with CAD analysis was applied to precisely access the cross-sectional areas of tested samples. With regard to a pooled group, apparent density for the coronal and sagittal cut plane was equal 1.53 g/cm^3^ and 1.57 g/cm^3^, tensile Young’s modulus 2.36 GPa and 2.14 GPa, and ultimate tensile strength 12.66 MPa and 14.35 MPa, respectively. No significant statistical differences (*p* > 0.05) were found for all the analyzed parameters when comparing coronal and sagittal plane cut groups. These observations confirmed the hypothesis that direction of sample cut does not affect the mechanical response of the orbital wall tissue, thus suggesting that mechanical properties of orbital wall bone show isotropic character.

## Introduction

The definition of the blowout fracture (known also as the expanded orbital fracture or isolated orbital floor fracture) was firstly proposed in 1950 by Converse and Smith [1]. The research covered characteristic kind of fracture of an intact bone margin and a distant range of destruction. The consequences may be functional disorders of the visual apparatus, aesthetic deformity and dysfunction of the maxillary nerve to possibly yield impairment of daily life aspects e.g. working or driving. The progress of civilization and increasing population activity leads to the growth of orbital fracture frequency. The orbital wall fractures are mostly caused by traffic accidents, fights, accidents at work or during sports activity, it mostly occurs at the age of forty [2]. Orbital wall fractures are significant diagnostic and therapeutic problems. Management of these fractures is a challenge; it requires a multidisciplinary approach. A highly specialized team to complete the task consist of maxillofacial surgeons, ophthalmologists, neurosurgeons, anesthesiologists and otolaryngologist surgeons [3]. Computer analysis and simulation are current tools to help the medical staff deal with this type of trauma [4–6].

To provide accurate computer modelling, three basic issues should be considered: (1) precise geometry of the considered region, (2) physical and mechanical properties of the human hard/soft tissues in the region, and (3) parameters of the applied loading (time variant magnitude and direction). The lattert is difficult to measure and predict as most of the blowout fractures are accidental. In this case, the computer simulations of various impact angles and magnitudes are essential [7,8]. The detailed geometry of human body parts and density information is available from computer tomography (CT) scanning, at present accompanying most surgical treatments. Their great benefit is their origin: living, healthy patients, skip any post-mortem alterations including both hard and soft tissues [5]. Several software packages automatically analyze scans and provide appropriate 3D numerical models [9,10]. Finally, the most challenging issue is precise identification of material properties of human tissues. In for the light of strength analysis, mechanical material parameters are substantial, so mechanical properties of different human body parts were experimentally tested throughout the years. Up till now, most of the bones are documented in detail, they are human femur, vertebrae, tibia [11,12], ribs [13,14] or outer skull base [15]. The authors suggest that the state-of-the-art database on properties of the orbital wall bones available in the literature is not complete, many authors confirm the need for development in the field [5,16]. For the sake of numerical computations, the properties of the orbital wall are averaged from the adjacent large bones of the skull, comprehensively verified before, e.g. like mandible and maxilla [17] or frontal and parietal skull bones [18]. However, this approach provides approximation of the real properties of the orbital wall, thinner and more fragile than other bone. It is unclear which model is right, isotropic or orthotropic. Moreover, in most finite element modeling (FEM), the skull bone is assumed isotropic and homogenous [19,20], to make the computation easier and faster.

Taking the above into consideration, in the present study it was decided to experimentally investigate the orbital wall bones under tension. The present study was aimed at following: (1) identify elastic modulus and ultimate tensile strength of orbital wall bones, (2) verify the specimen plane cut impact on the orbital wall tissue strength response. Deep comprehension of the orbital wall tissue mechanics is essential to the finite element models of the eyeball and orbit trauma. This action is bound to improve: (a) operation surgery techniques, (b) preparation of 3D printing post-traumatic implants, and (3) treatment guidelines of eye trauma-related illnesses.

## Materials and methods

### Sample preparation

**Fig 1:**
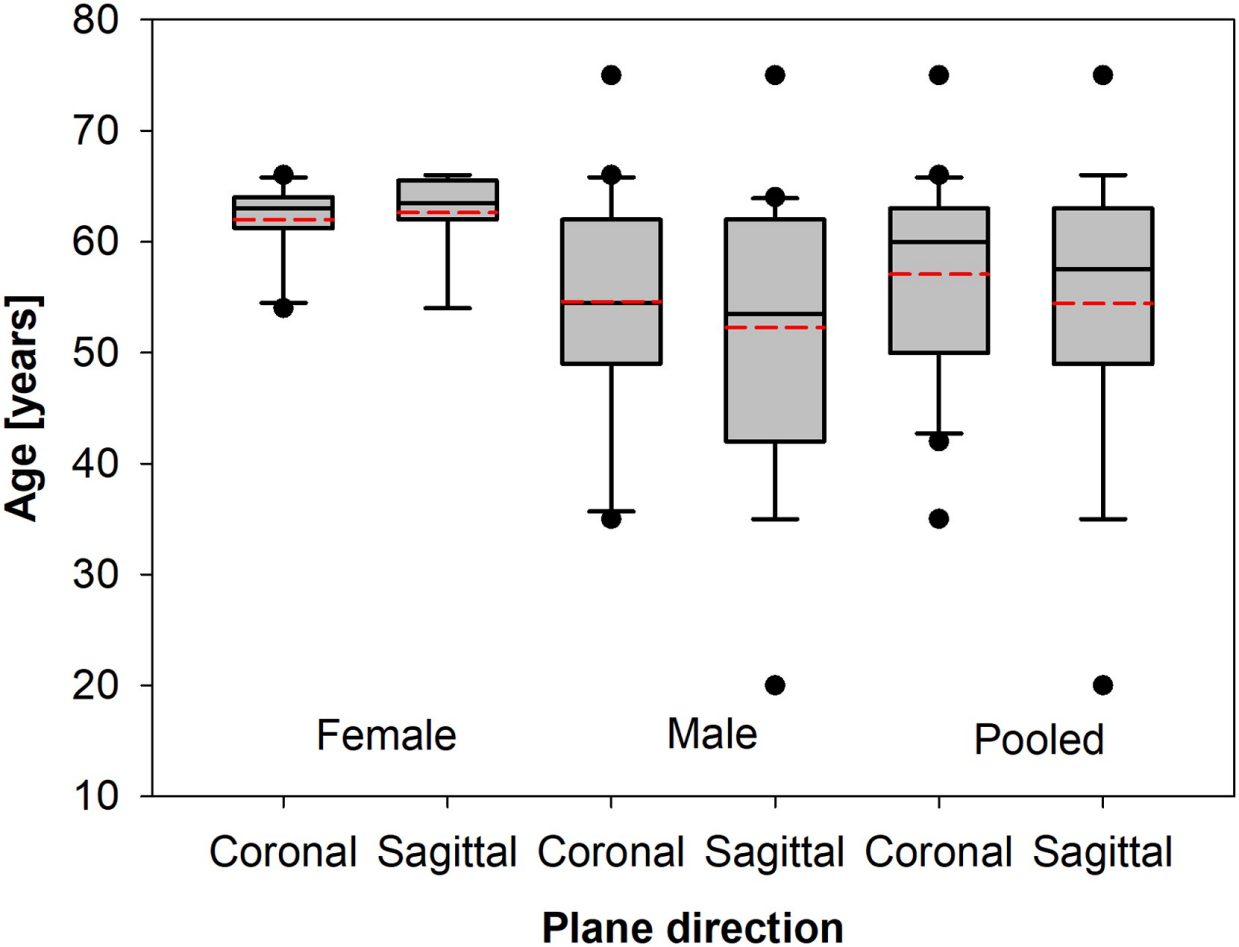
Box plots of age differences between particular gender groups and with a split for the coronal and sagittal plane cut.

The consent of the Independent Bioethics Committee of the Medical University of Gdańsk was obtained for the realization of the presented research on human bone material (NKBBN521-565/2020). The investigation was performed according to Helsinki Convention recommendations [21] and the European Union’s Directive 2004/23/EC art. 13,15 [22]. All human specimens were acquired from cadavers during a medico-legal autopsy performed no later than 2–5 days after their death. Cadavers were participants of fatal sudden accidents, therefore only informed verbal consent was obtained from their families for the use of clinical data, when possible. The biological material was collected from 33 different cadavers (aged between 20 to 80 years old, 21 males and 12 females). The age distribution of patients tested in the study is shown in Fig 1.

Only the patients that had not suffered from any chronic diseases or mechanical head injuries were selected for sample preparation. The specimens of an orbital bone (without periosteum) were obtained directly after cranial cavity opening and brain removal for histological routine examination. Next the specimens were cut off from the superior and medial walls of the orbit as a single integral bone piece. The bone samples were collected from both orbits or a single one, right or left, with regard to bone quality and operating possibilities. These bone blocks (all in the length range 30–40 mm) were put into the 0.9% NaCl at a temperature -20°C for the next 6 to 36 hours, prior to laboratory tests. The sample preparation method was similar to the procedures proposed before by Morgan et al. for experiments on human vertebra, tibia and femur [11]. Before mechanical laboratory testing each bone was defrosted to the temperature of about 20°C and then cut with surgical clippers either alongside the coronal or the sagittal plane to form repetitive specimens 7–15 mm wide, 0.7–2.3 mm thick and 30–40 mm long. In the sample preparation, course special care was taken by the otolaryngologist surgeon to get the straight-shaped samples in the greatest possible extent. Finally, some bone blocks were broken during sample preparation or testing machine mounting, while in some cases it was possible to get even three samples from one block. There was also a key rule, that from the left orbit the only prepared samples were taken in the coronal plane, while from the right orbit the only samples were cut along the sagittal direction. As a result, more than 100 samples were prepared for testing. However, some specimens fractured during placing in machine grips, these samples were excluded from the analysis. Sometimes right after the force was applied some samples cracked, so sugesstion arose that additional bending complements axial force in the specimen. These samples were also excluded from the analysis. Finally, 68 samples were successfully tested and taken into account for analysis.

### Testing procedure

The mechanical tests were performed in the laboratory of Structural Mechanics, Department at the Faculty of Civil and Environmental Engineering at Gdansk University of Technology (Gdansk, Poland). Prior to testing, every specimen was dried with a paper towel, next weighted and its volume was determined by the method based on Archimedes principle. The specimen apparent density was computed as follows:

$$\rho_{\text{app}}=\frac{\text{bone}\;\text{tissue}\;\text{weight}}{\text{gross}\;\text{volume}}$$ (1)

**Fig 2:**
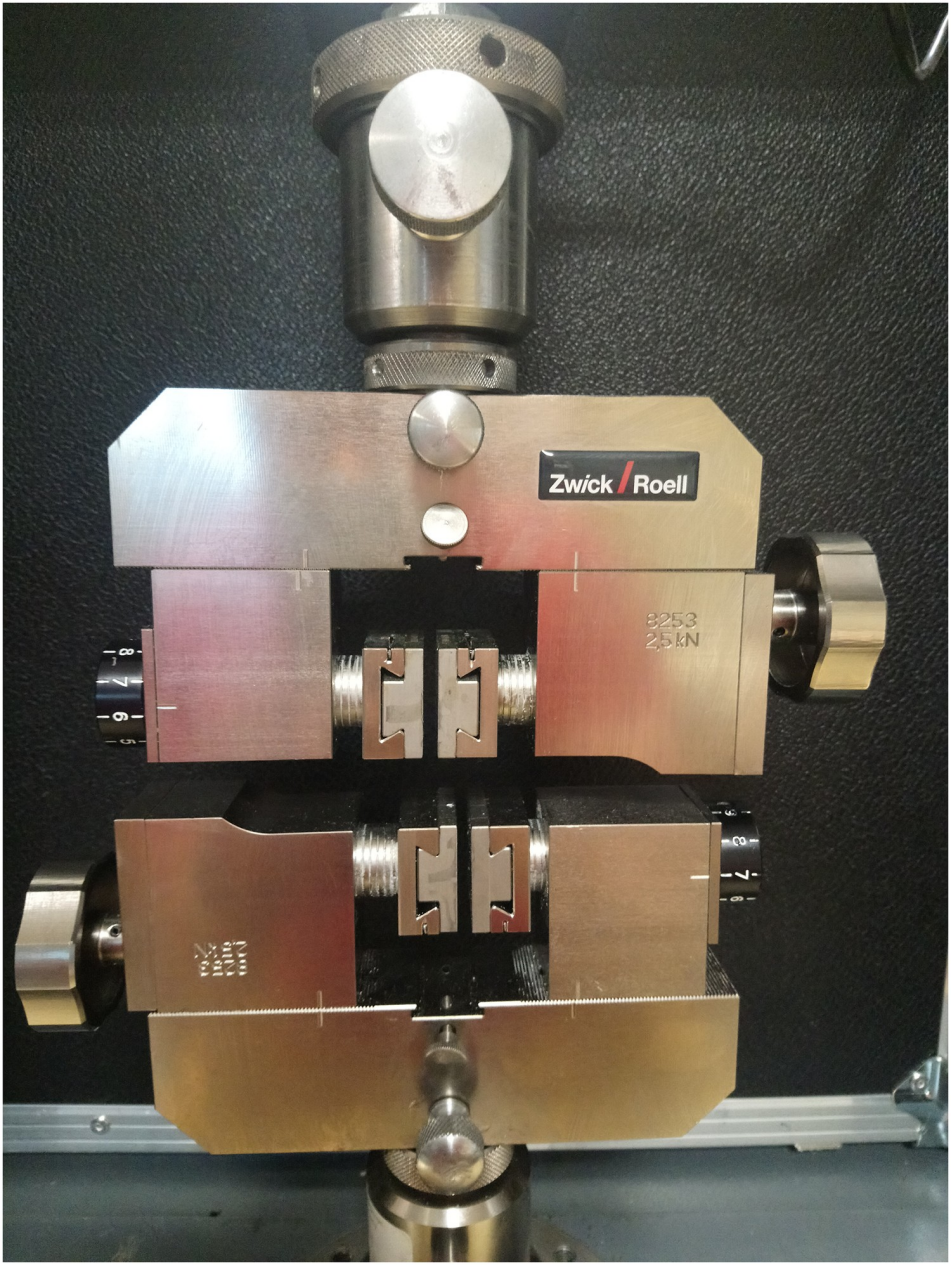
Grips dedicated for asymmetrically shaped samples.

**Fig 3:**
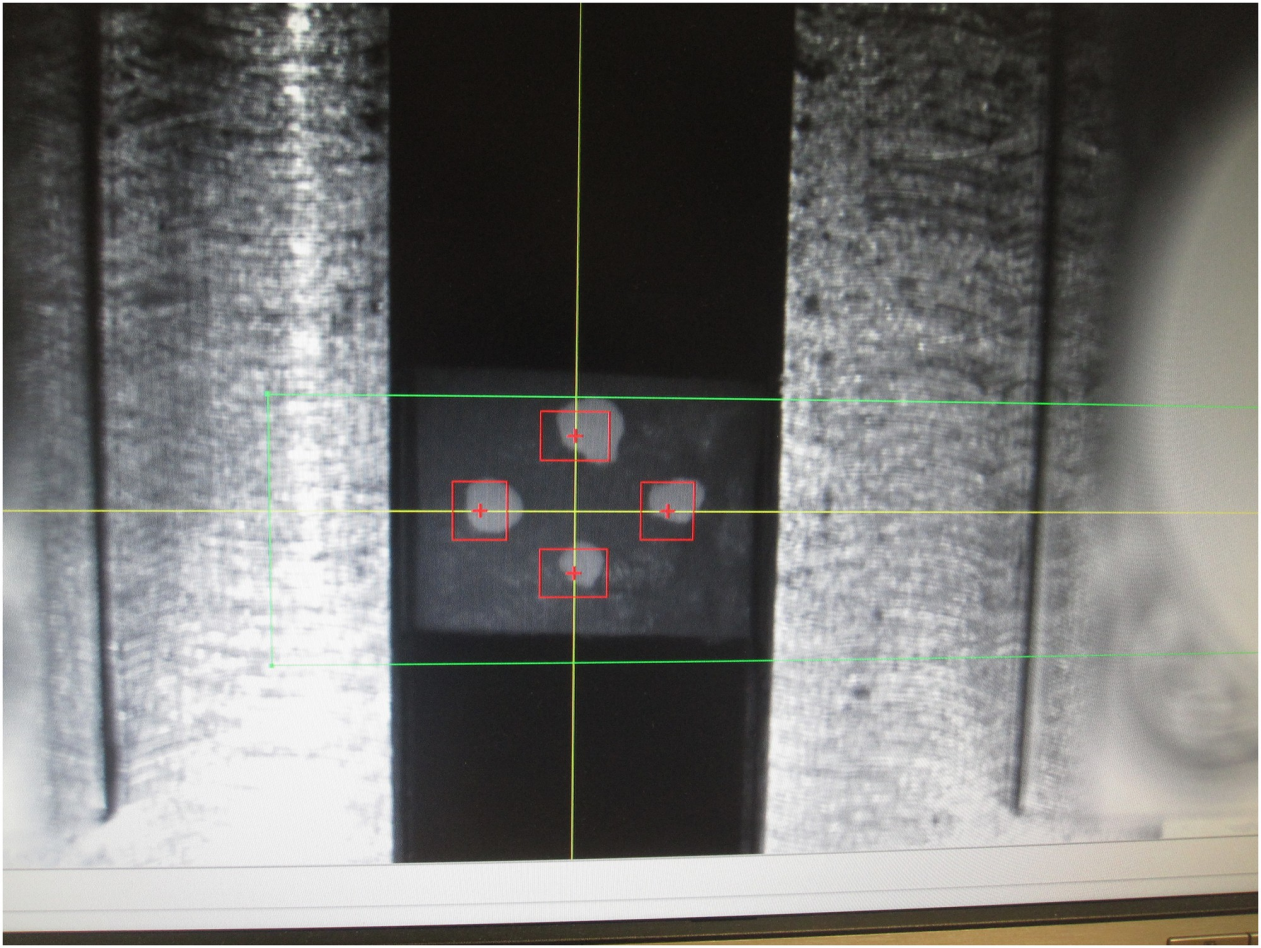
White markers followed by the video extensometer during test realization.

Uniaxial tensile tests untill failure with 0.01 mm/s crosshead rate were performed on the Zwick Roell Z0/20 testing machine. Special machine grips dedicated to asymmetric, nonlinear-shaped samples were applied for tight and stable positioning of the bone fragments at the testing machine (Fig 2). The sample curvature was relatively uniform along the tension direction, thus if the sample did not break at the preparation stage or the test start, it was considered correctly positioned, fulfilling the requirements for uniaxial tension tests. Before a test each sample was marked with four white dots, followed by the video extensometer during the test to exactly register the specimen deformation (Fig 3). There was no need to touch the sample during the testing procedure, thus reducing the risk of erroneous results.

**Fig 4:**
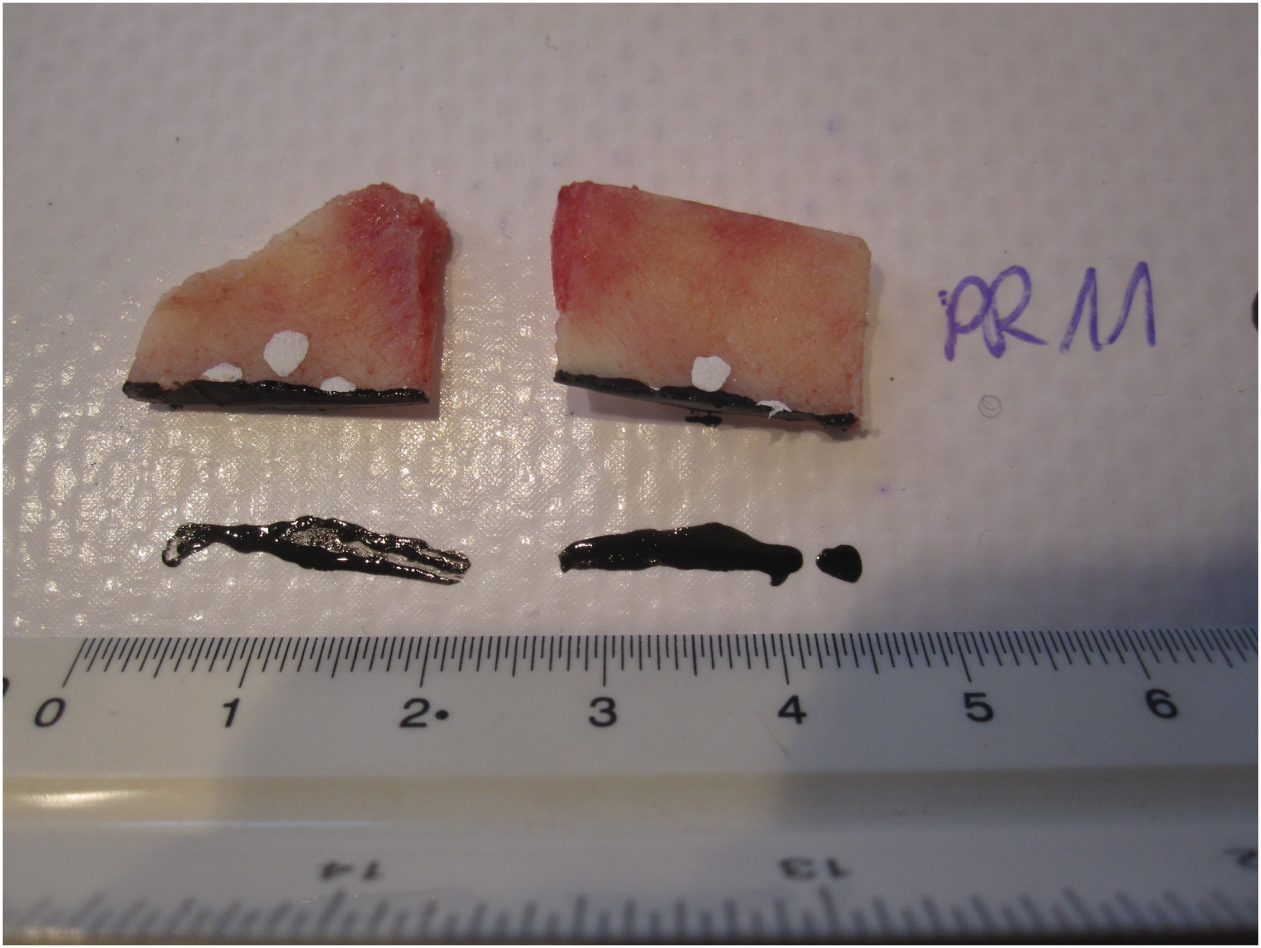
Specimen cut with the surgeon clippers and cross-section of both parts imprinted by ink on non-soaking technical fabric material.

The preload of 10 N was applied to eliminate the mounting errors, the crosshead rate during the test was set at 0.01 mm/s. The tests were performed up to specimen rapture or to the force level fall by 20%. The tensile force and load-elongation curves were automatically registered by the TestXpert II software dedicated to Zwick/Roell testing machine. In the experimental course small plastic effects were observed only, so it was assumed that the cross-sectional area remained constant before and after the test. After the test, each specimen was cut with surgical clippers near the fracture spot, the cut edges were polished with sandpaper (P400) and the cross-section of both parts of the specimen was carefully imprinted with ink on the non-soaking technical fabric material (Fig 4). High-resolution photographs of the footprints were taken to be later imported to the AutoCAD system, here the area of each imprint was assessed, following a given scale (Fig 4). Making the footprint results more credible, the mean of the left and right cross-sectional areas of the same specimen was taken in the further analysis as the final cross-sectional area of a particular specimen.

### Young’s modulus identification procedure

**Fig 5:**
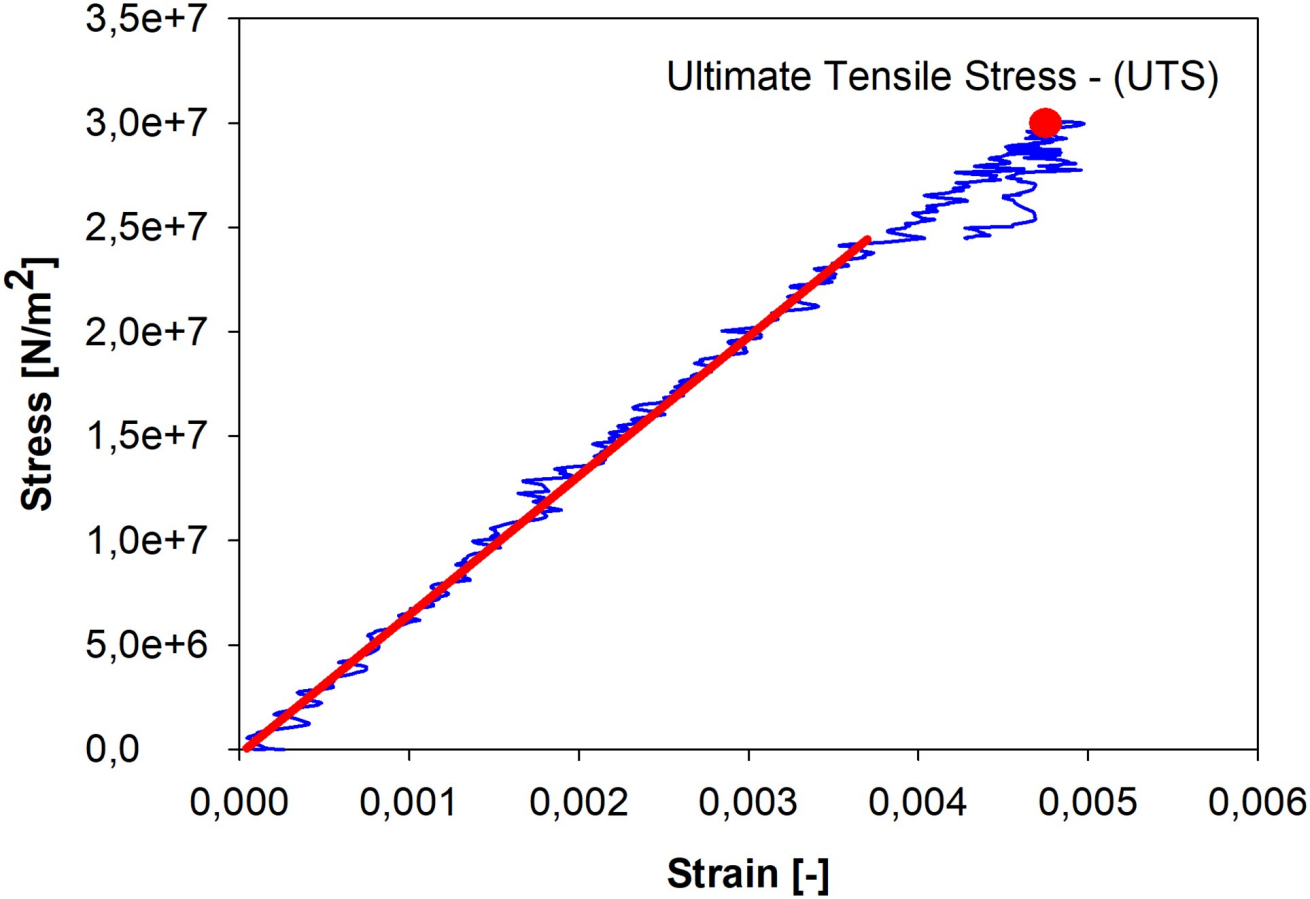
Typical stress-strain curve obtained for orbital wall bone. The red line indicates the linear part of the curve and its slope corresponds to Young’s modulus.

The data registered by TestXpert operating the testing machine during mechanical tests and cross-sectional areas from the imprinting method were applied as the input in the SigmaPlot software to compute the cross stress and strain values. Firstly, the stress *σ = F/A* was taken in its simple form as the force *F* acting on the cross-sectional area *A*. The strain *ε* = Δ*L*/*L*_0_ was taken an engineering definition as the change in distance between two longitudinal extensometer markers Δ*L*, related to the initial distance *L*_0_ between these markers. The obtained results were plotted in the graph form of the stress-strain relation curve (blue line in Fig 5). This curve can be approximated by a linear function at the whole range. It confirms small impact of plastic effects here. The linear part of the graph, defined by a function (red line in Fig 5) was identified by regression supported by the least-squares method. The approximation was performed in Sigmaplot 12.0 software. The coefficients of determination (R^2^) of the performed least-squares approximation were always higher than 0.95. The slope of the linear part was defined as tensile Young’s modulus (*E-modulus*), marking the elastic property of the tested sample. The ultimate tensile strength (UTS), defined as the greatest stress level before the failure of the sample, was also reported.

### Statistical evaluation

The statistical evaluation of the obtained results was conducted by the SigmaPlot 12.5 software. All the results were compared between the coronal and sagittal plane cuts within the female and male groups, next the female and male groups were pooled together. In some cases it was possible to get three samples from a single human orbit, but sometimes a single sample from one orbit was taken only. To provide statistical analysis each sample was taken as an individual measurement. The normal distribution of parameters for each group was assessed by the Shapiro-Wilk test. To individually compare groups of normal distribution, the t-Student test was used, in the case of non-normal distribution the Mann-Whitney U test was applied. Each time the significance level was p < 0.05.

## Test results

**Fig 6:**
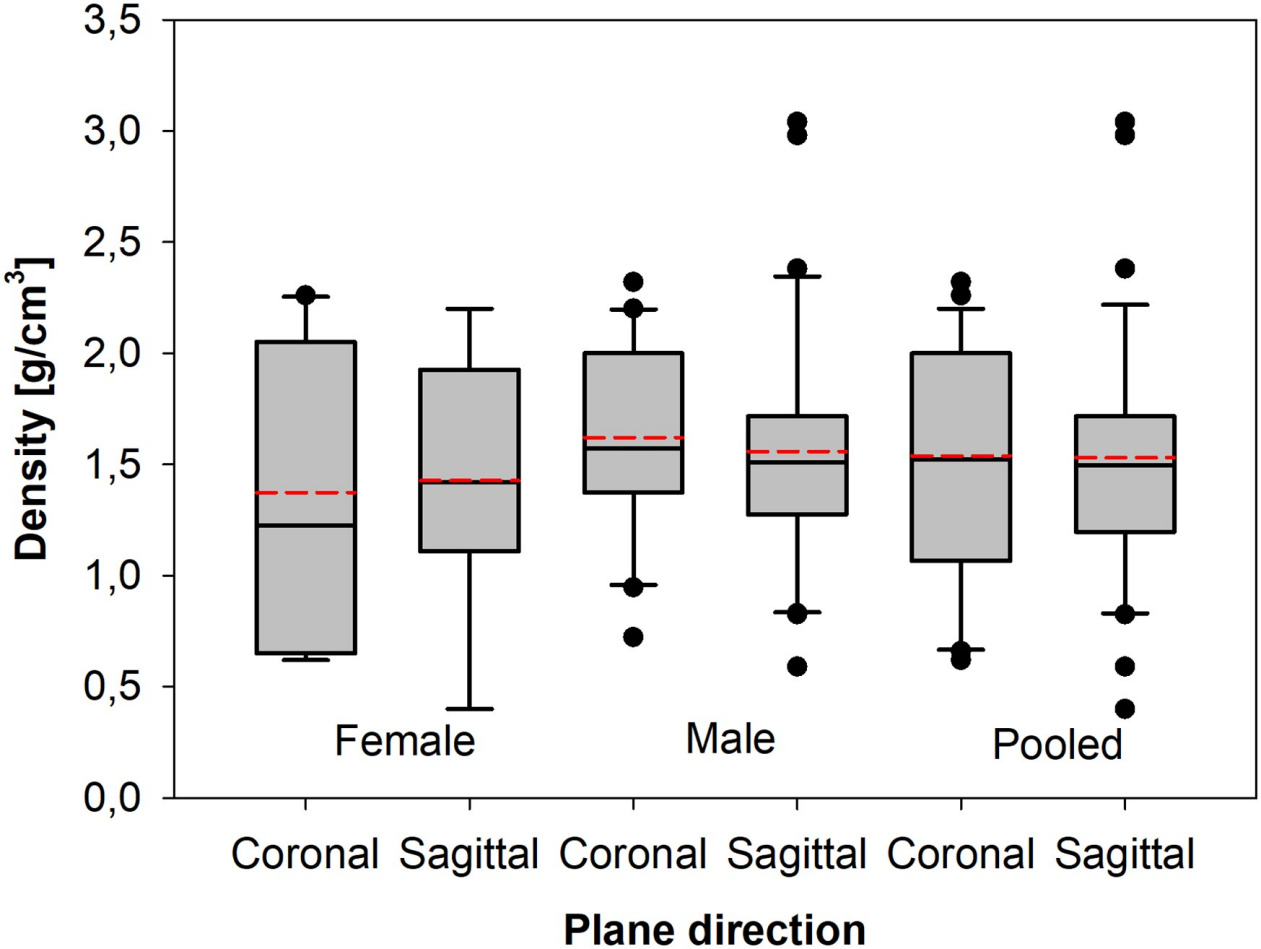
Box plots of density differences between particular gender groups and with a split for the coronal and sagittal plane cut.

**Fig 7:**
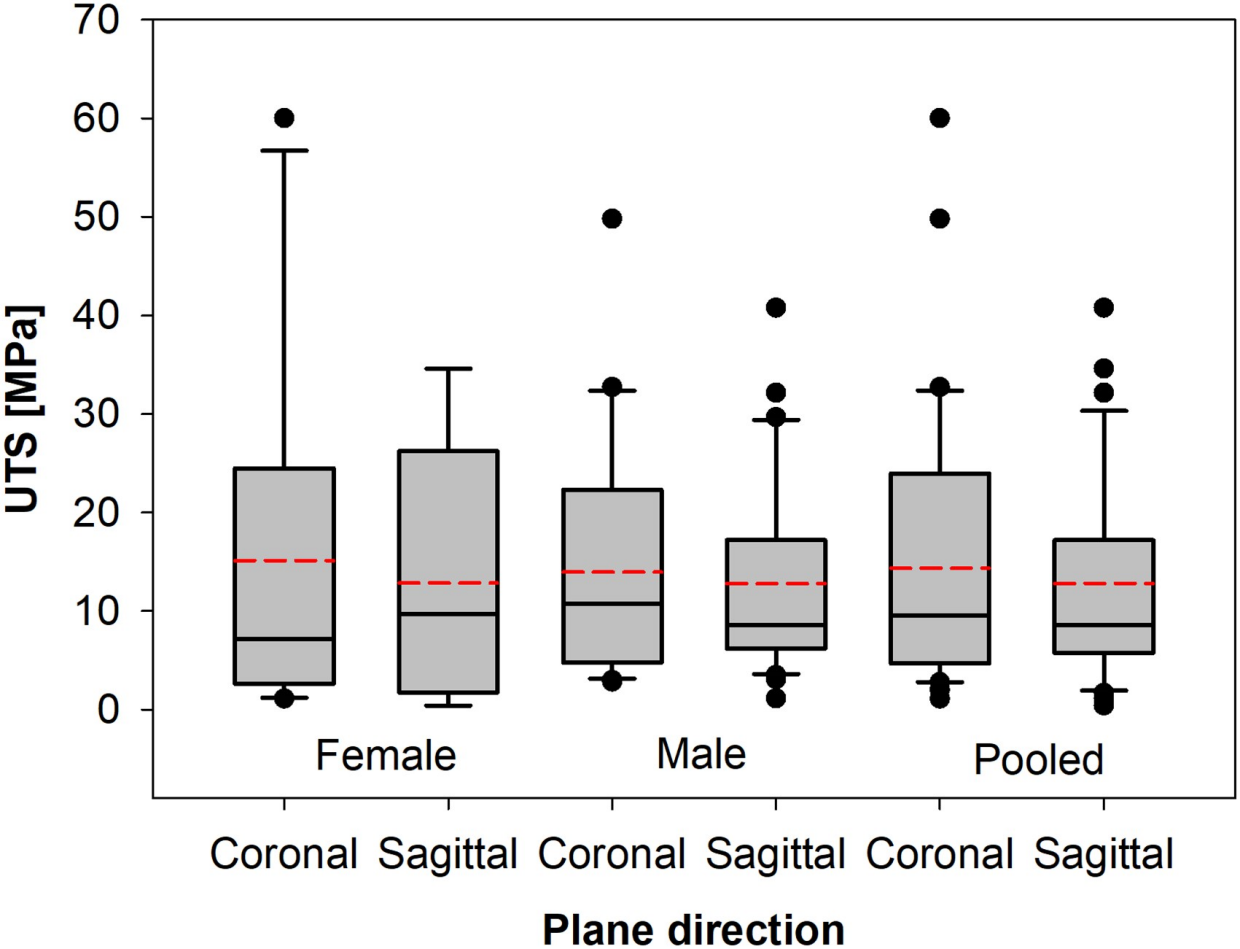
Box plots of ultimate tensile stress (UTS) differences between particular gender groups and with a split for the coronal and sagittal plane cut.

**Fig 8:**
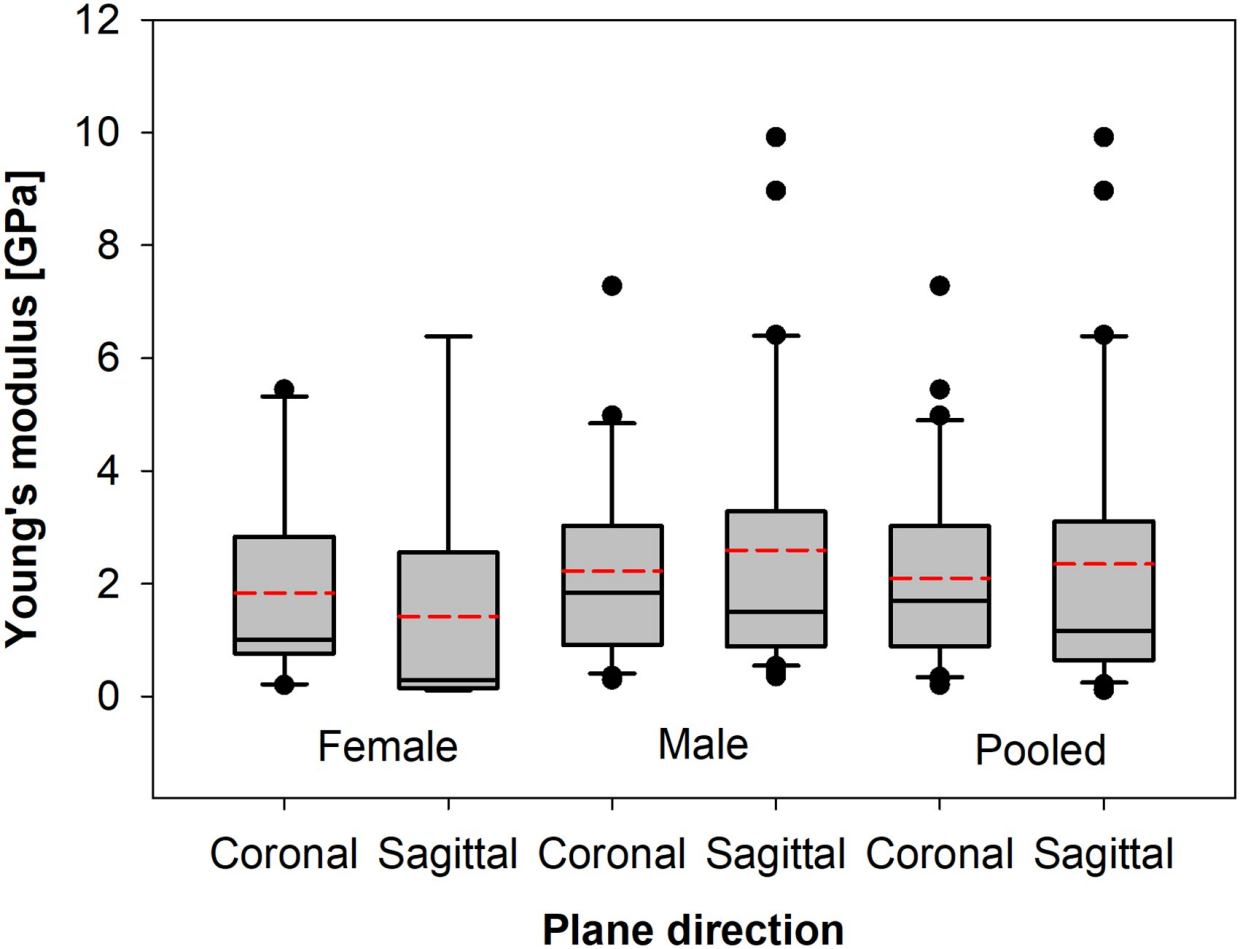
Box plots of tensile (Young) modulus differences between particular gender groups and with a split for the coronal and sagittal plane cut.

This study was principally performed to investigate material properties of the orbital human wall bone concerning the coronal and sagittal planes of the orbital wall. The biological material was collected from 33 different cadavers (aged between 20 to 75 years old, 12 females and 21 males), finally, the total number of samples successfully tested and identified was *N* = 68 (18 females, 50 males). The collection results, i.e. elastic tensile modulus (*E*-*modulus*), ultimate tensile stress (UTS) and apparent density (*ρ*_*app*_) is presented in Table 1. The groups are compared in the form of box plots (Figs 6–8), here the box represents the values between the first and third quartile, the horizontal black line is the median, the red line is the mean value, the whiskers mark the minimum and maximum values, finally, the black dots indicate the outlier values of the particular set of data. The density distribution for the pooled results (sagittal and coronal plane cut) follows the normal distribution for male and female groups No statistically significant difference occurs (t-Student test p = 0.952) between the apparent density 1.58 g/cm^3^ for the male group and 1.40 g/cm^3^ for the female group (sagittal and coronal plane cut pooled). It confirms the correctness of the performed measurements, as the bone density of a particular gender group is similar, regardless of the cut direction (Fig 6).

**Table 1:** Identification results of all tested specimens.

	Sagittal plane			Coronal plane			Pooled (sagittal+coronal plane)		
	*E-modulus* [GPa]	*UTS* [MPa]	*ρ*_*app*_ [g/cm^3^]	*E-modulus* [GPa]	*UTS* [MPa]	*ρ*_*app*_ [g/cm^3^]	*E-modulus* [GPa]	*UTS* [MPa]	*ρ*_*app*_ [g/cm^3^]
Male	2.23 (*N* = 20) *M*: 1.84 *R*: 0.29÷ 7.28 *SD*: 1.69 *IQR*: 0.91÷2.23	*14*.*0* (*N* = 20) *M*: 10.8 *R*: 2.83÷ 49.80 *SD*: 12.49 *IQR*: 4.79÷22.30	1.62 (*N* = 20) *M*: 1.57 *R*: 0.72÷ 2.32 *SD*: 0.44 *IQR*: 1.38÷2.00	2.60 (*N* = 30) *M*: 1.51 *R*: 0.36÷ 9.91 *SD*: 2.55 *IQR*: 0.89÷1.72	12.8 (*N* = 30) *M*: 8.59 *R*: 1.14÷ 40.77 *SD*: 9.73 *IQR*: 6.19÷17.2	1.56 (*N* = 30) *M*: 1.51 *R*: 0.55÷ 3.04 *SD*: 0.55 *IQR*: 1.28÷1.72	2.45 (*N* = 50) *M*: 1.63 *R*: 0.30÷ 9.91 *SD*: 2.23 *IQR*: 0.92÷3.05	13.26 (*N* = 50) *M*: 8.92 *R*: 1.14÷ 49.80 *SD*: 10.8 *IQR*: 5.30÷17.20	1.58 (*N* = 50) *M*: 1.53 *R*: 0.59÷ 3.04 *SD*: 0.50 *IQR*: 1.30÷1.84
Female	1.84 (*N* = 10) *M*: 1.01 *R*: 0.20÷ 5.44 *SD*: 1.73 *IQR*: 0.76÷2.84	*15*.*14* *(N = 10)* *M*: 7.21 *R*: 1.12÷ 60.02 *SD*: 18.2 *IQR*: 2.60÷24.51	1.37 *(N = 10)* *M*: 1.23 *R*: 0.62÷ 2.26 *SD*: 0.66 *IQR*: 0.65÷2.05	1.42 *(N = 8)* *M*: 0.29 *R*: 0.15÷ 6.39 *SD*: 2.25 *IQR*: 0.15÷2.25	12.86 *(N = 8)* *M*: 9.71 *R*: 0.43÷ 34.60 *SD*: 13.1 *IQR*: 1.76÷26.3	1.43 *(N = 8)* *M*: 1.42 *R*: 0.40 ÷2.20 *SD*: 0.57 *IQR*: 1.11÷1.93	1.65 *(N = 18)* *M*: 0.93 *R*: 0.12÷ 6.39 *SD*: 1.93 *IQR*: 0.24÷2.57	14.13 *(N = 18)* *M*: 8.31 *R*: 0.43÷ 60.02 *SD*: 15.73 *IQR*: 2.01÷24.51	1.40 *(N = 18)* *M*: 1.41 *R*: 0.40 ÷2.26 *SD*: 0.60 *IQR*: 0.96÷2.00
Pooled (Female+Male)	2.14 (*N* = 30) *M*: 1.70 *R*: 0.20÷ 7.28 *SD*: 1.70 *IQR*: 0.89÷3.02	14.35 (*N* = 30) *M*: 9.56 *R*: 1.12÷ 60.02 *SD*: 14.33 *IQR*: 4.70÷23.98	1.57 (*N* = 30) *M*: 1.53 *R*: 0.62 ÷ 2.32 *SD*: 0.49 *IQR*: 1.07÷2.00	2.36 (*N* = 38) *M*: 1.17 *R*: 0.11÷ 9.91 *SD*: 2.51 *IQR*: 0.64÷3.11	12.66 (*N* = 38) *M*: 8.59 *R*: 0.07÷40.77 *SD*: 10.47 *IQR*: 5.75÷17.20	1.53 (*N* = 38) *M*: 1.50 *R*: 0.40÷ 3.04 *SD*: 0.55 *IQR*: 1.18÷1.72	2.24 (*N* = 68) *M*: 1.46 *R*: 0.12÷ 9.91 *SD*: 2.17 *IQR*: 0.82÷3.03	13.49 (*N* = 68) *M*: 8.90 *R*: 0.43÷60.02 *SD*: 12.18 *IQR*: 5.00÷17.60	1.53 (*N = 6*8) *M*: 1.50 *R*: 0.40÷ 3.04 *SD*: 0.53 *IQR*: 1.19÷1.90

*N*–number of samples.

*E-modulus–*Young’s modulus [GPa].

*UTS*–Ultimate Tensile Strength/Stress [MPa].

*ρ*_*app*_*−*apparent density [g/cm^3^].

*R*–range.

*M*–median value.

*SD*–standard deviation.

*IQR*–interquartile range Q1÷Q3.

Elastic tensile modulus (*E*-*modulus*) and ultimate tensile stress (UTS) for the coronal, sagittal and pooled coronal+sagittal cut plane groups failed to the Gaussian criterion of the Shapiro-Wilk test (*p* < 0.050).

There was no statistically significant difference (UTS: Mann-Whitney U test p = 0.835, E-modulus: Mann-Whitney U test p = 0.913) between coronal and sagittal plane cuts for the male group.

No statistically significant difference (UTS: Mann-Whitney U test p = 0.824, E-modulus: Mann-Whitney U test p = 0.143) was detected between coronal and sagittal plane cuts for the female group.

There was no statistically significant difference (UTS: Mann-Whitney U test p = 0.810, E-modulus: Mann-Whitney U test p = 0.697) between coronal and sagittal plane cuts for the pooled male and female groups.

## Discussion

The head bones are heterogeneous in their size, structure and function. Various studies on the Young’s modulus range for different head bones were carried out [23]. Verschueren et al. [24] completed three-point bending tests on the 191 cranial bone specimens prepared from 118 skulls, both frozen and fresh, aimed at the bicycle helmet research. The results showed Young’s moduli ranging from 1.6 to 2.5 GPa for frozen specimens, 2.3 to 5.4 GPa for fresh specimens. However, the specimens were taken from the parietal area of the head only. A similar approach was completed by Motherway et al. [25] and Auperrin et al. [26], who tested 63 and 351 cranial bone samples, respectively. All of the specimens were acquired from various locations of 8 and 21 skulls, respectively. The total thickness of cranial bones, apparent density and apparent elastic modulus from dynamic 3 point-bending tests were reported. Additionally, in the study of Motherway et al. [25], all the specimens were scanned using μCT and various dynamic test speeds regarded. The Young’s moduli and loading rates were 7.46±5.39 GPa (0.5 m/ s), 10.77±9.38 GPa (1.0 m/s) and 15.54±10.29 GPa (2.5 m/s). In the study by Auperrin et al. [26] study, the average apparent flexural modulus among different skull bones were 3.81±1.55 GPa for the frontal bones, 5.00 ±3.12GPa for parietal bones and 9.70 ±5.75GPa for temporal bones. However, these researches did not include other bones building the skull, limiting the study to the cranial bones only.

Seong et al. [17] applied the nanoindentation technique to find that Young’s modulus differs significantly between the maxilla (14.9 GPa) and mandible (18.3 GPa), as well as between posterior and anterior parts of the jaws (17.5 GPa versus 15.7 GPa). They also found statistical correlation between Young’s modulus, hardness and apparent density. Yan et al. [27] indicated that the value of the skull Young’s modulus is primarily determined by the properties of the cortical bone (13 GPa), while modulus of trabecular bone is lower (0.89–1.00 GPa) [27].

In the case of small flat bones that build the orbital bone walls, it was usually assumed for the numerical FEM analysis that Young’s modulus covers the range 11.8 GPa to 15.4 GPa [8,28,29], in other reports 11 GPa [5]. However, these tests were usually performed on dry skulls or based only on generally fixed values for other bones. The authors of the present paper built two FE models of the human orbital region to simulate the buckling mechanism of orbital wall damage. Numerical computation was conducted using the results of preliminary research of the flat bones obtained from medial and superior orbital wall parts, here Young’s modulus was estimated at 1.3 GPa [7].

Advantages and disadvantages of the performed experimental tests should be noted. The curvature of the tested samples sometimes led to their fracture during placing them in the grips of the testing machine. To solve that problem, special grips dedicated to non-symmetrical, non-linear samples were installed. Their special shape and the possibility of precise clamping adjustment allowed for accurate sample placement in the Zwick/Roell Z0/20 testing machine. Firstly, more than 100 samples were prepared for testing, but finally, 68 underwent mechanical testing successfully, thus contributing the base of the presented results.

The bone samples were cut out of the spherical-shaped orbital human wall, therefore it was almost impossible to get regular and repetitive sample shapes. Hence, the footprint technique was introduced to measure the cross-sectional area of each specimen individually and introduce it in modulus assesment. The cross-sectional area of a particular specimen was taken as the mean of the ink stamps from both parts of the cut sample. The water non-absorbable technical fabric was used as the stamping surface to avoid errors made by ink splash on paper or similar easily soaking materials. The greatest advantage of the stamping technique in the analysis of orbital wall bones is its ability to exlude empty spaces and sinus presence in the specimens, thus giving true cross-sectional areas of the tested samples.

The results obtained in this work cover the limits of the results of other authors. It should be emphasized that the diffference between the presented Young’s modulis and the measurements of jaw bones (maxilla, mandible), bones protecting the central nervous system (skull cap) or supporting bones (eg. femurs, vertebrae) can be significant. It may result from a different structure of the orbital bones and from their location inside the cranium, in the latter case they are exposed to direct impacts in a lower extent. Even in the case of closely located bones, performing similar functions–e.g. mandible and jaw, as well as bone parts, various moduli values were found of statistically significant differences [12,17]. The orbit is structured from the eyeball, intraorbital structures, bone and the closest neighborhood. The anatomical structure determines its function: mostly the bone structure, the presence of adipose tissue and other structures (e.g. eyeball, ligaments and muscles, nerves, arteries) is important. Thus, the greatest impact on increasing strength and resistance to injury is detected for orbital roof thickness (about 15% per 1 mm) and orbital floor thickness (about 20% per 1 mm) [30]. The presence of adipose tissue inside the orbit also causes a different effect. Due to its presence, the orbit is prone to compression. There is a possibility of orbit deformation as of the absorption result of energy released as a result of trauma. It confirms the protective role of the orbit. The orbital wall, which provides the shape and resistance to injury, shows a multimodal and complicated structure. In addition to bony structures, it is formed by connective tissue structures—periorbita. Both these tissues adhere tightly to each other near the outer rim and the top of the orbit, while in other places their connection is rather loose. The histological structure of both tissues, the ratio of hard (osteocytes) to soft (connective tissue cells) elements, their vascularization, the presence of both natural openings (channels for vessels, nerves, congenital dehiscences) and those associated with ongoing or past pathological processes (inflammation, neoplasm, trauma) determine their strength and other physical and chemical properties. Changing with age the stroma of both tissues, hormonally conditioned chemical composition is decisive for elasticity and resistance of trauma. The presence of collagen fibers in the stroma, growing from a well-circulated periosteum into the bone (Sharpey’s fibers), acts strongly on the orbit’s integrity, however, their arrangement is quite irregular [31]. The elastic modulus of the periosteum is almost 30 times greater than its value for the bones that make up the orbit [27,32]. The orbital bone resistance to injury is related to its unique shape in the entire human anatomy—a truncated cone, which increases its resistance to injury [33,34]. Summing up, no difference in Young’a modulus and UTS for different sample cut planes may result from irregular composition of collagen fibers building orbital wall tissue, bringing more isotropic mechanical properties of this tissue, identified and reported in the study. To get the most accurate test results of the current study the authors used several solutions to improve the quality of measurements:

1. special grips dedicated to non-symmetrical samples were introduced for mechanical testing;
2. the non-contact measuring system of the video-extensometer applied for displacement registration;
3. the ink footprint technique coupled with CAD analysis was applied to precisely determine of cross-sectional areas of tested samples.

Another innovative solution introduced to the presented research is the method of sample taking—orbital bones were cut in two directions: sagittal plane (always from the right orbit) and coronal plane (always from the left orbit). It allowed to compare the results and evaluate the hypothesis that specimen plane cut affect the orbital wall bone mechanical response. The ultimate tensile stress and Young’s modulus did not show statistical differences while compared within a group of a particular plane cut direction of the samples. This observation suggests that the investigated orbital wall bones show similar mechanical properties in both sagittal and coronal planes. Based on the current research, it seems reasonable to focus on a larger number of samples of a more homogeneous age distribution. The impact of age on mechanical properties of orbital wall bones may be also analysed. These further tests and analysis are necessary to make a reliable human eye orbit model for more detailed studies on different orbital bone damage mechanisms related to blow-out traumas. It is a development towards the state–of-the-art base on blow-out traumas to improve the following: (a) operation surgery techniques, (b) preparation of 3D printed implants for craniofacial surgeries and (3) treatment guidelines of eye trauma-related illnesses.

## Supporting information

S1 File (XLSX)
